# Induction of type II collagen expression in M2 macrophages derived from peripheral blood mononuclear cells

**DOI:** 10.1038/s41598-022-25764-4

**Published:** 2022-12-15

**Authors:** Fu-Hui Wang, Chia-Ying Hsieh, Ching-I. Shen, Chang-Han Chuang, Yu-Hsuan Chung, Chi-Chung Kuo, Kuan-Der Lee, Chih-Lung Lin, Hong-Lin Su

**Affiliations:** 1 Duogenic StemCells Corporation, Taichung, Taiwan; 2 Department of Orthopedics, Show Chwan Memorial Hospital, Changhua, Taiwan; 3 National Chung Hsing University, Taichung, Taiwan; 4 Rong Hsing Research Center for Translational Medicine, National Chung Hsing University, Taichung, Taiwan; 5 Department of Neurology, Taichung Tzu Chi Hospital, Buddhist Tzu Chi Medical Foundation, Taichung, Taiwan; 6 School of Post-Baccalaureate Chinese Medicine, Tzu Chi University, Hualien, Taiwan; 7 Department of Medical Research and Cell Therapy and Regenerative Medicine Center, Taichung Veterans General Hospital, Taichung, Taiwan; 8 Department of Medicine and Center for Cell Therapy and Regenerative Medicine, College of Medicine, Taipei Medical University, Taipei, Taiwan; 9 Department of Neurosurgery, Asia University Hospital, 222, Fuxin Rd., Wufeng Dist., Taichung City, Taiwan; 10 Department of Occupational Therapy, Asia University, Taichung, Taiwan; 11 Department of Life Sciences, National Chung Hsing University, 145, Xin-Da Rd., South Dist., Taichung City, 402 Taiwan

**Keywords:** Biological techniques, Biotechnology

## Abstract

The human type II collagen (Col II), specifically expressed in chondrocytes, is a crucial component of the adult hyaline cartilage. We examine the potential of artificial induction of Col II in human peripheral blood mononuclear cells (PBMNCs) as a novel Col II provider. Human PBMNCs were purified and were treated with high doses of macrophage-colony stimulating factor (M-CSF), granulocyte macrophage-colony stimulating factor (GM-CSF), or granulocyte-colony stimulating factor (G-CSF) and examined the Col II expression at indicated days. Quantitative Col II expression was validated by real-time reverse transcriptase-polymerase chain reaction (RT-PCR), immunocytochemistry, and flow cytometry. We demonstrate that monocytes in PBMNCs can be artificially induced to express both Col II proteins and M2 macrophage markers by the high concentration of colony-stimulating factors, especially M-CSF and GM-CSF. The Col II proteins were detected on the cell membrane and in the cytoplasm by flow cytometry and immunocytostaining. Combination with IL-4 provided a synergistic effect with M-CSF/GM-CSF to trigger Col II expression in M2 macrophages. These CD206 and Col II double-expressing cells, named modified macrophages, share M2 macrophages' anti-inflammatory potency. We demonstrated that the modified macrophages could significantly attenuate the inflammatory progress of Complete Freund's adjuvant (CFA)-induced arthritis and collagen-induced arthritis in rodents. Here, we provide the first evidence that a modified macrophage population could ectopically express Col II and control the progress of arthritis in animals.

## Introduction

Osteoarthritis (OA), the most common musculoskeletal disease, is characterized by progressive degeneration of the articular cartilage with synovial inflammation (synovitis) and neovascular invasion into the articular surface^1^. The gradual wear and tear of cartilage bring local inflammation of the joint lining and surrounding soft tissues. Rheumatoid arthritis (RA), another common chronic joint disease, affects 0.5–1.0% population and involves in autoimmune dysregulation^1^. RA affects multiarticular cartilages, bones, synoviums, and sometimes internal organs. Severe OA and RA both are accompanied by progressively subchondral bone destruction, osteophyte formation, bone marrow remodeling, and meniscal lesions.

Accumulative evidence suggests that macrophage plays a crucial role in developing both OA and RA^2,3^. Macrophages are the primary immune cells and the front-line responder to joint injury in the normal synovium^3,4^. In the OA synovium, macrophages cause cartilage destruction by producing matrix metalloproteinases and inflammatory cytokines, such as IL-1β and TNF-α^5^. Increased macrophages in the affected synovium are correlated with the degree of synovial angiogenesis and synovitis^3,6,7^. A recent clinical study by Kraus et al. showed that the numbers of activated macrophages are positively associated with the OA and RA severity and the disease progression^8^.

It is known that macrophages exhibit a spectrum of diverse functions and phenotypes. To elucidate the detailed role of the macrophage in arthritis, Bailey et al. locally depleted the intra-articular macrophages in genetically modified mice with joint injury. They surprisingly discovered that such depletion leads to enhanced synovial inflammation and M1 polarization after fracture^9^. This controversial result may be caused by the diverse functionally-distinct macrophage lineages, such as M1 and M2 macrophages. M1 macrophages are pro-inflammatory and can be polarized by granulocyte macrophage-colony stimulating factor (GM-CSF). In contrast, M2 macrophages are anti-inflammatory and can be polarized by macrophage colony-stimulating factor (M-CSF) to reduce synovitis. Depletion of macrophages in animal models of arthritis may lead to M2 macrophage reduction in the synovial membrane and subsequently trigger the synovitis progress^9^. These findings in genetically-edited mice suggest that polarized M1/M2 macrophages from monocytes are critical for arthritis progress and could be essential biomarkers in arthritis management.

The human type II collagen (Col II) is a crucial but non-renewable component of the adult joint cartilage^10,11^. Providing exogenous Col II stimulates the secretion of cartilage glycosaminoglycans (GAGs) by chondrocytes and enhances cartilage repair in animal models^12,13^. However, Col II shows very limited turnover during the life span of an adult, even in pathological arthritis condition^10,11^. Current OA therapies include pain relief with oral NSAIDs or selective cyclooxygenase 2 (COX-2) inhibitors and intra-articular (IA) injections with corticosteroids or hyaluronan^14^. Partial or total joint replacement is the final solution for severe OA and RA patients. Although hyaluronan is used to lubricate damaged-joint, this treatment does not regenerate hyaline cartilage or modify disease progression^14^. Despite advances in understanding the biology of arthritis and other musculoskeletal diseases, developing disease-modifying arthritis drugs, such as biological molecules and novel cell therapy, is still an urgent unmet medical need for promoting the healing of damaged joints.

## Results

**Figure 1:**
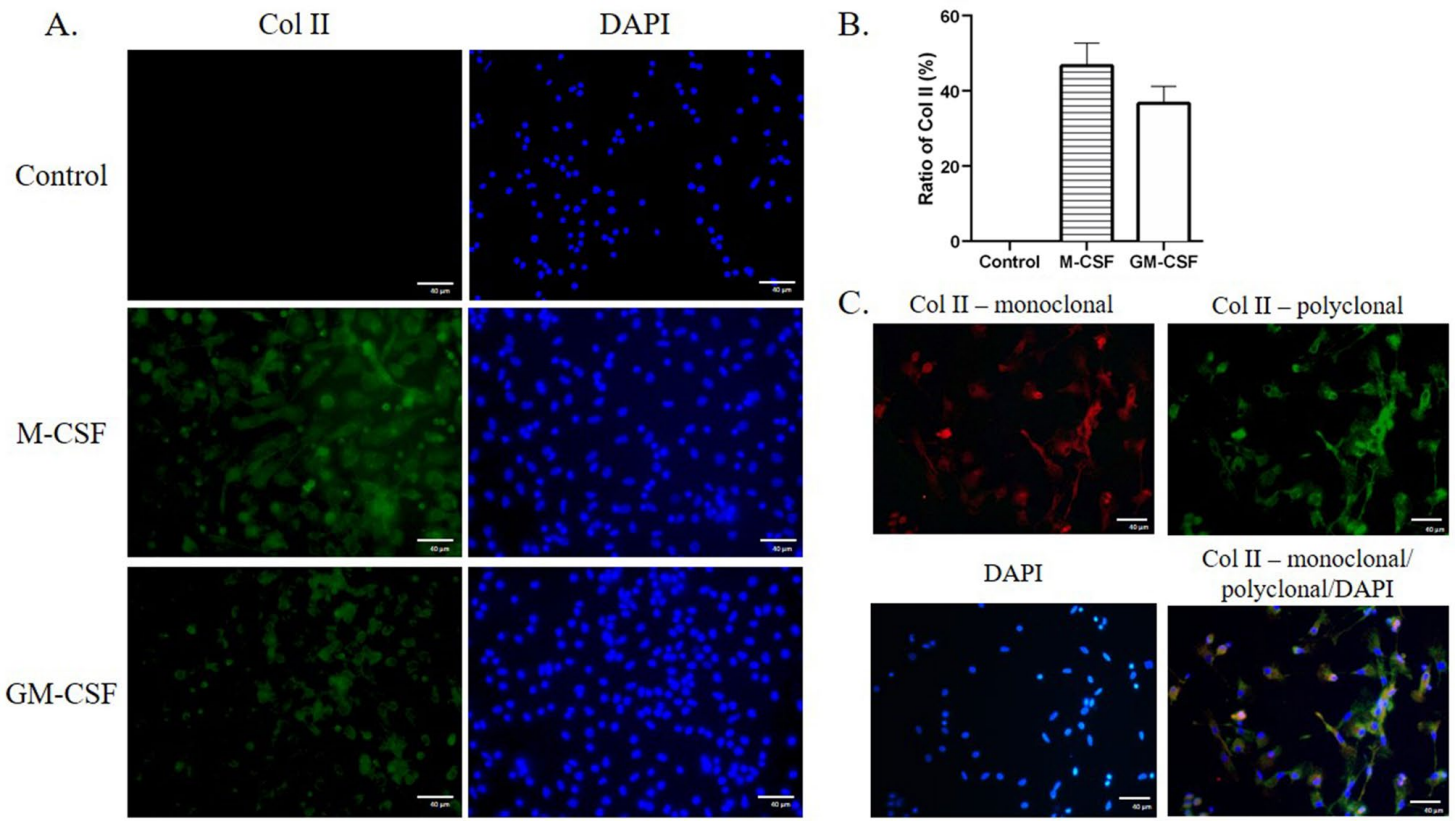
The Col II expression in the differentiating PBMNCs. The cultured PBMNCs were adherent on fibronectin-coated plates and treated with 100 ng/mL cytokines, including the M-CSF, GM-CSF, or G-CSF for 3 days. The Col II expression (green) in the cultured PBMNCs was stained with an anti-human Col II polyclonal antibody (**A**), and the percentage of Col II expressing cells was summarized from triplicated experiments (**B**). The fidelity of Col II expression was further confirmed by a specific monoclonal antibody against human Col II (**C**). The nuclei were stained with DAPI as blue.

Peripheral blood samples were collected from donors and subjected for isolating the peripheral blood mononuclear cells (PBMNCs) by Ficoll-Paque premium. After treating PBMNCs with growth factors for 3 days on fibronectin-coated dishes, we showed that 100 ng/mL M-CSF steered 46.7 ± 6.0% adherent PBMNCs to express Col II in the cells, detected by a polyclonal antibody (Fig. 1A,B). Similar Induction efficacy of GM-CSF was observed (36.7 ± 4.5%) (Fig. 1A,B). A Col II-specific monoclonal antibody further verified the Col II expression in adherent PBMNCs (Fig. 1C). Both antibodies showed positive and similar cytosolic expression patterns after fluorescent immunocytochemistry staining (Fig. 1C).

**Figure 2:**
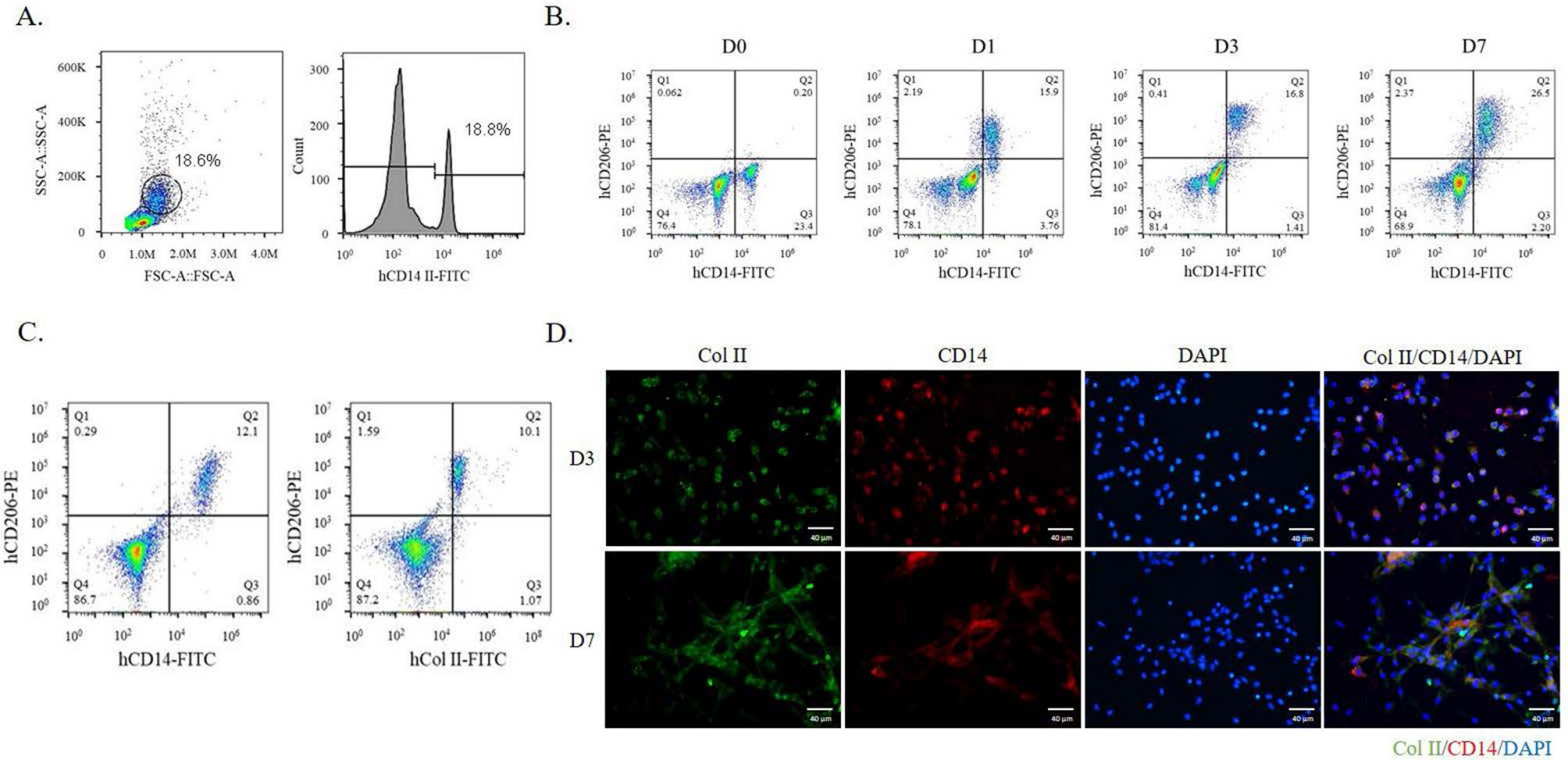
The Col II expression in M2 macrophages. The monocyte population of PBMNCs was identified either by flow cytometry of FSC/SSC or CD14-FITC histogram (**A**). The M2 population was verified by both CD14 (monocyte-specific marker) and CD206 (M2-specific marker) expressions at indicated days (**B**). The coexpression of CD206 and Col II in CD14^+^ monocytes was detected on Day 5 culture (**C**). The presence of CD206 indicates the cultured CD14^+^ monocytes have matured into modified macrophages. The M-CSF-treated PBMNCs on day 3 (D3) and day 7 (D7) were immunostained with Col II (green) and CD14 (red) (**D**). The nuclei were stained with DAPI as blue.

To quantify the Col II expression and examine the Col II under suspension culture, we examined the percentage of monocyte in the PBMNCs by flow cytometry using both anti-CD14 antibody and anti-Col II antibody. The population of monocytes could be determined by both CD14 expression or the size distribution in the plot of forward scatter (FSC), and side scatter (SSC) (Fig. 2A). To investigate the cell fate of Col II expressing cells, we co-stained the living PBMNCs with anti-CD14, anti-CD206, or anti-Col II antibodies on days 1, 3 and day 7 to identify the Col II expressing cells. CD206 is a specific M2 macrophage surface marker. With 100 ng/mL M-CSF treatment, CD14^+^ monocytes were polarized to become CD14^+^/CD206^+^ M2 macrophage at a ratio of 15.9%, 16.8% and 28.5% on day 1, day 3 and day 7, respectively (Fig. 2B). The flow cytometry further reveals a unique subgroup in the M2 macrophage population, named modified macrophage in this invention, which expressed Col II, CD14, and CD206 proteins on the cell membrane at day 3 (Fig. 2C). The results were further confirmed by the immunocytostaining of Col II and CD14 in the adherent PBMNCs (Fig. 2D).

**Figure 3:**
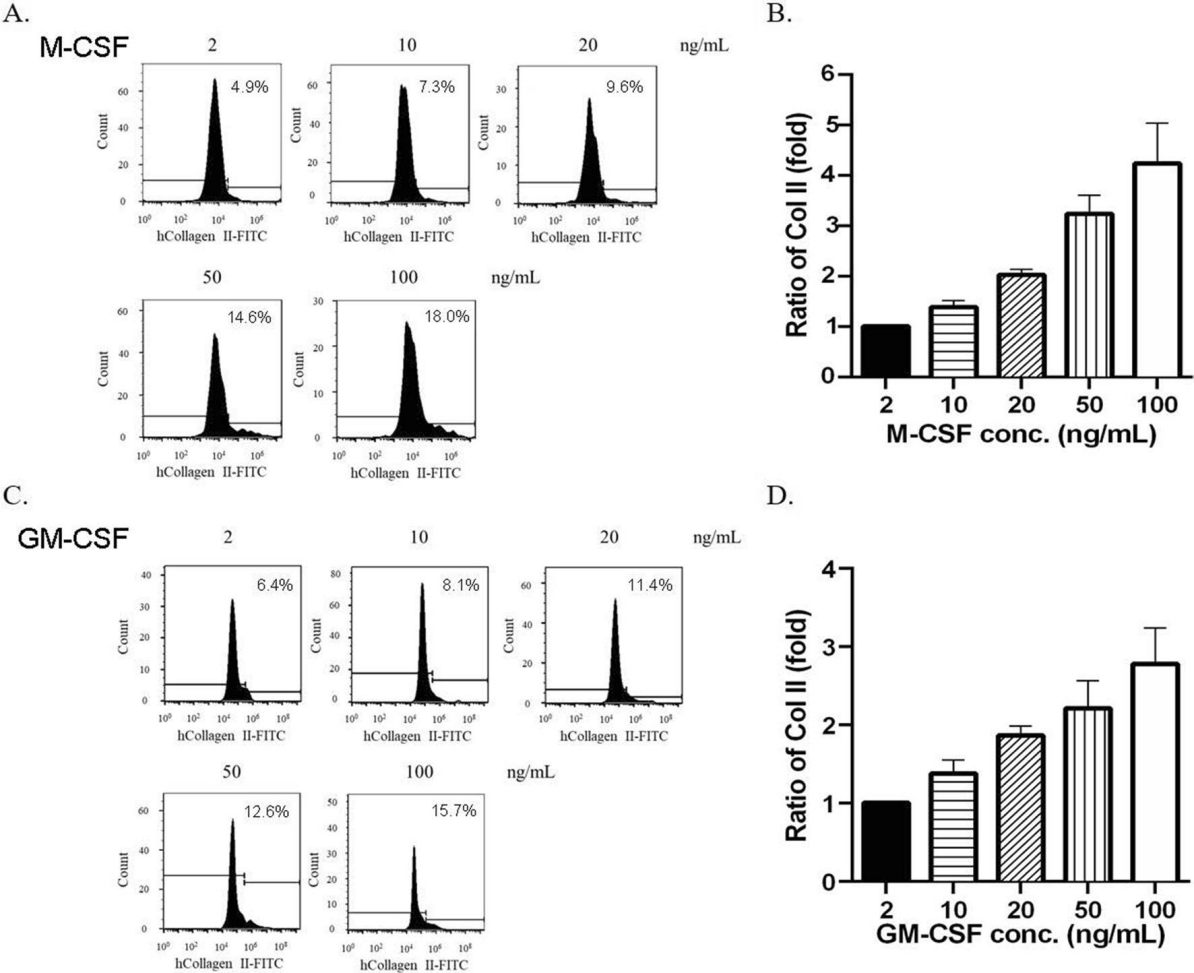
The dose effects of M-CSF (**A**, **B**) and GM-CSF (**C**, **D**) on Col II expression of modified macrophages were examined by flow cytometry on day 3. The fold induction results (**B**, **D**) were analyzed from triplicated experiments.

We next examine the dose–effect of M-CSF/GM-CSF on Col II expression. The PBMNCs were treated with 2, 10, 20, 50, and 100 ng/mL M-CSF (Fig. 3A) or GM-CSF (Fig. 3B) for 3 days. Cell populations were first gated based on the FSC-A and SSC-A to identify monocytes, and the monocyte population was further analyzed based on Col II expression. The result of Fig. 3 showed that 10 ng/mL M-CSF or 10 ng/mL GM-CSF, the typical concentrations for macrophage polarization, only activated about 5% Col II-expressing cells. Higher doses of the cytokines, such as 100 ng/mL M-CSF and GM-CSF, efficiently steered the modified macrophage induction in a dose-dependent manner (Fig. 3B and D).

**Figure 4:**
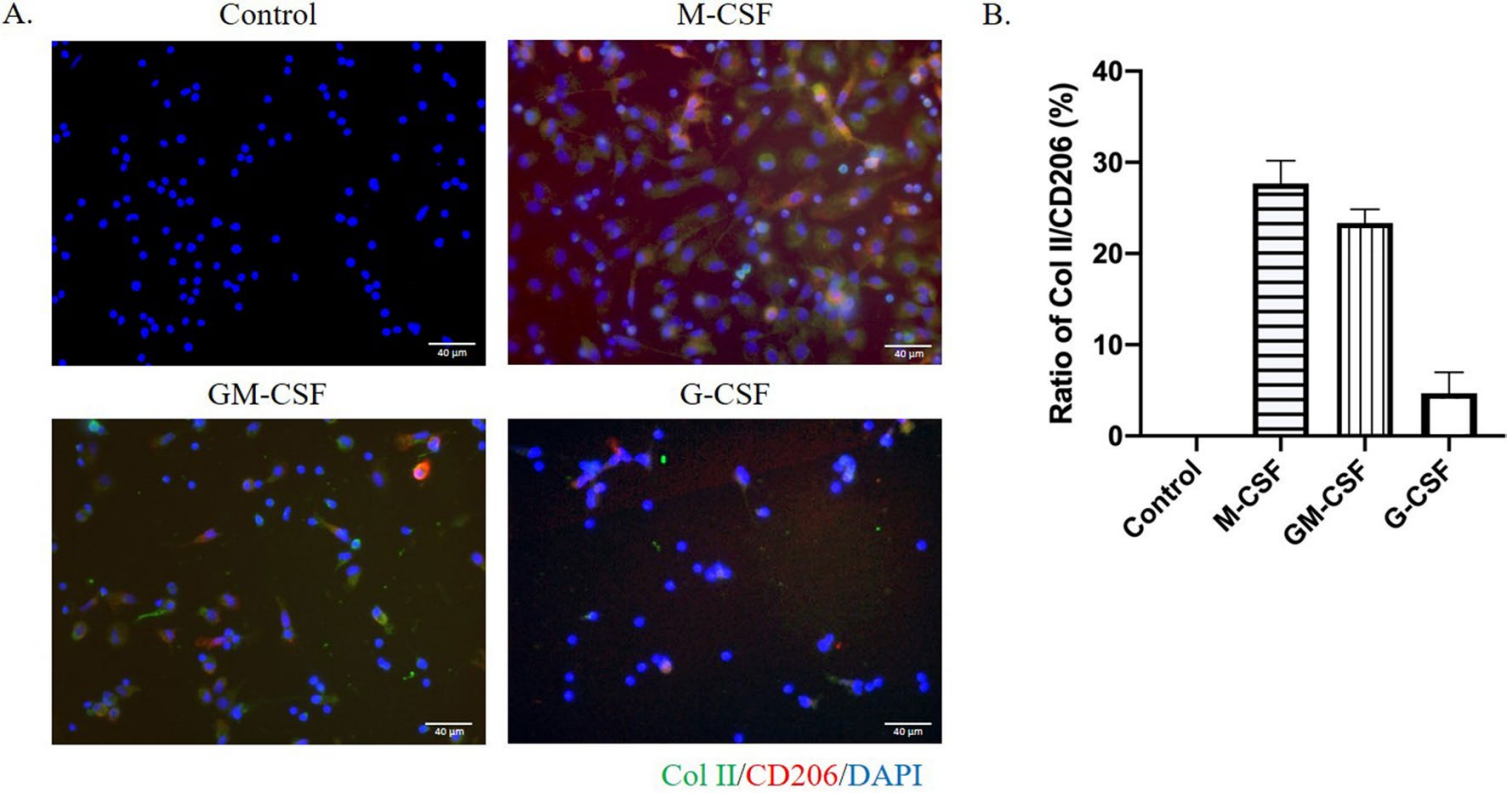
The effect of 100 ng/mL M-CSF, GM-CSF, and G-CSF on the induction of Col II (green) and CD206 (red) coexpression in modified macrophages (**A**). The ratio of Col II expression in CD206^+^ cells was analyzed by Image J from three independent immunocytostaining results (**B**).

In contrast to the M2 polarizing factor of M-CSF, GM-CSF is a M1 polarizing factor and shows lower M2 induction efficacy. The treated PBMNCs with either control medium, M-CSF, GM-CSF, or G-CSF, were cultured in suspension for 3 days and then adhered on fibronectin-coated slides. Interestingly, immunocytochemical staining results with anti-CD206 antibody (red) and anti-Col II antibody (green) identified the robust induction of modified macrophage by either M-CSF or GM-CSF treatments (lane 2, 27.7 ± 2.5% ; lane 3, 23.3 ± 1.5%; Fig. 4A,B), but not the control medium (lane 1, 0% , Fig. 4A,B) and G-CSF (lane 4, 4.7 ± 2.3%, Fig. 4A,B).

**Figure 5:**
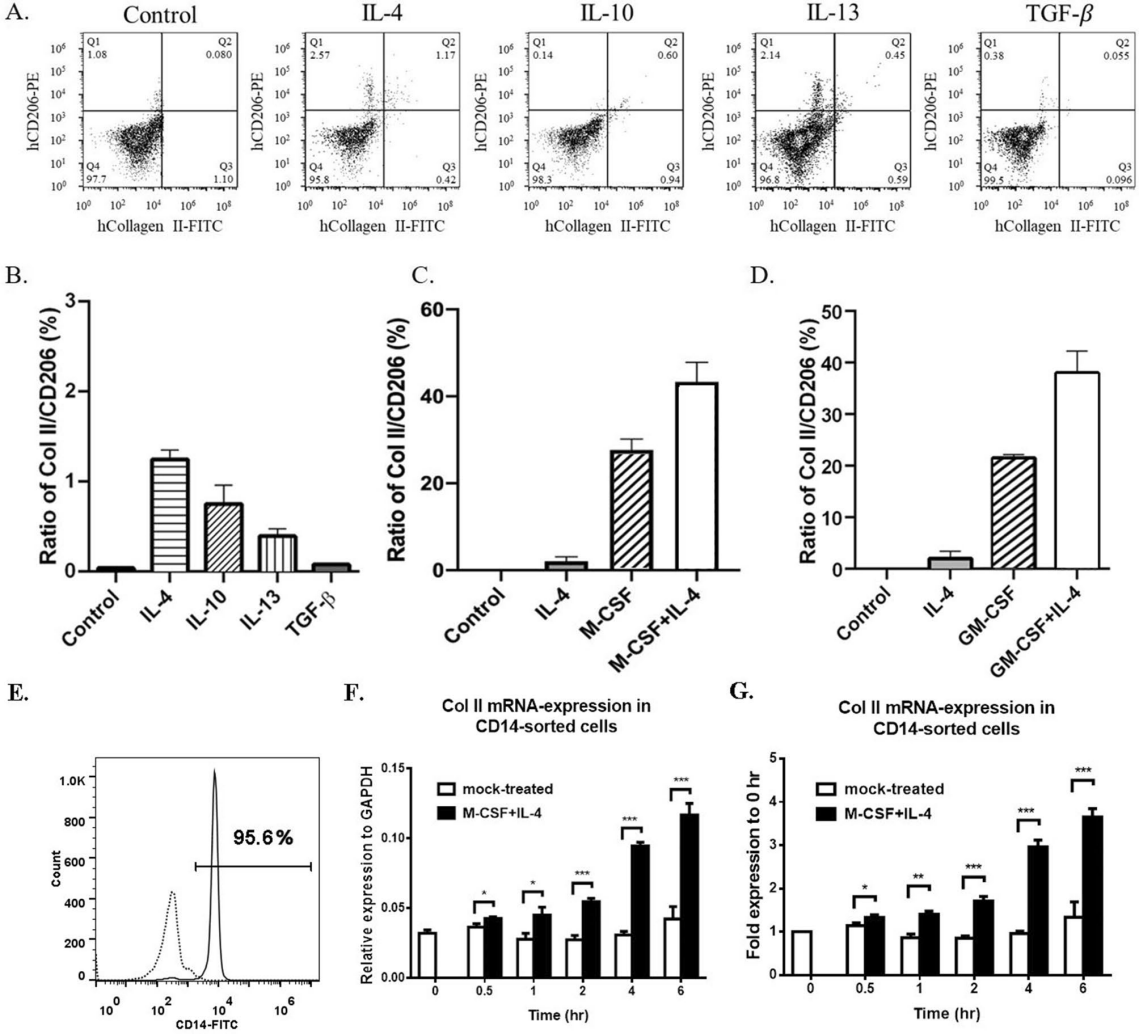
The effects of various cytokines on inducing Col II and CD206 expression of modified macrophages were examined by flow cytometry on day 3 (**A**, **B**). The ratio of Col II/CD206 modified macrophages of cultured PBMNCs was examined in the conditions of IL-4/M-CSF (**C**) and IL-4/GM-CSF (**D**), based on the results of the immunocytochemical staining on day 3. The Col II mRNA expression was analyzed by quantitative real-time RT-PCR at 0.5, 1, 2, 4, and 6 h after the addition of PBS (mock-treated) or M-CSF + IL-4 combination in the CD14 beads-enriched monocytes (**E**). The Col II mRNA expression was normalized to the housekeeping gene expression of glyceraldehyde 3-phosphate (GAPDH) (**F**) and shown as the fold difference at the indicated times (**G**). Statistic results are analyzed from three independent experiments. One-way ANOVA, *, *p* < 0.05; ** *p* < 0.01; ***, p < 0.001.

M2 macrophages can be initially induced by M-CSF from naïve monocytes and become mature by IL-4, IL-10, IL-13, or TGF-β. We next examined which mature factor can potentiate the M-CSF/GM-CSF effect on enforcing Col II expression. We isolated the PBMNCs and cultured the PBMNCs with control medium alone, IL-4, IL-10, IL-13, TGF-β at 20 ng/mL for 3 days. Cell populations were immunostained to identify modified macrophage populations based on Col II and CD206 expression. Our results indicated that among the tested cytokines, IL-4 showed the highest induction ratio of modified macrophage from the PBMNCs population (Fig. 5A,B).

We next examined the M-CSF/IL4 and GM-CSF/IL-4 combinations on Col II expression. The PBMNCs were cultured under four conditions, including control medium, 100 ng/mL IL-4, 100 ng/mL M-CSF or GM-CSF alone, 100 ng/mL M/CSF or GM-CSF with 100 ng/mL IL-4, for 3 days on fibronectin-coated slides. Compared to individual M-CSF (27.6 ± 2.5%) or IL-4 groups (2.0 ± 1.0%), M-CSF and IL-4 combinations showed synergistic induction (43.3 ± 4.5%, > 27.6 ± 2.5% + 2.0 ± 1.0%) on modified macrophages from the cultured PBMNCs (Fig. 5C). A similar synergistic result was observed for the GM-CSF and IL-4 combination (Fig. 5D).

Based on this optimized Col II induction protocol, we determined the Col II RNA expression profile in isolated monocytes during the early induction. Treating the CD14-sorted PBMNCs (> 90% purity, Fig. 5E) with M-CSF/IL-4 combination induced the Col II RNA expression in a time-dependent manner within the first 6 h culture (Fig. 5F,G). In the mock-treated cells, no significant difference in Col II RNA expression could be detected at the first 6 h of treatment.

**Figure 6:**
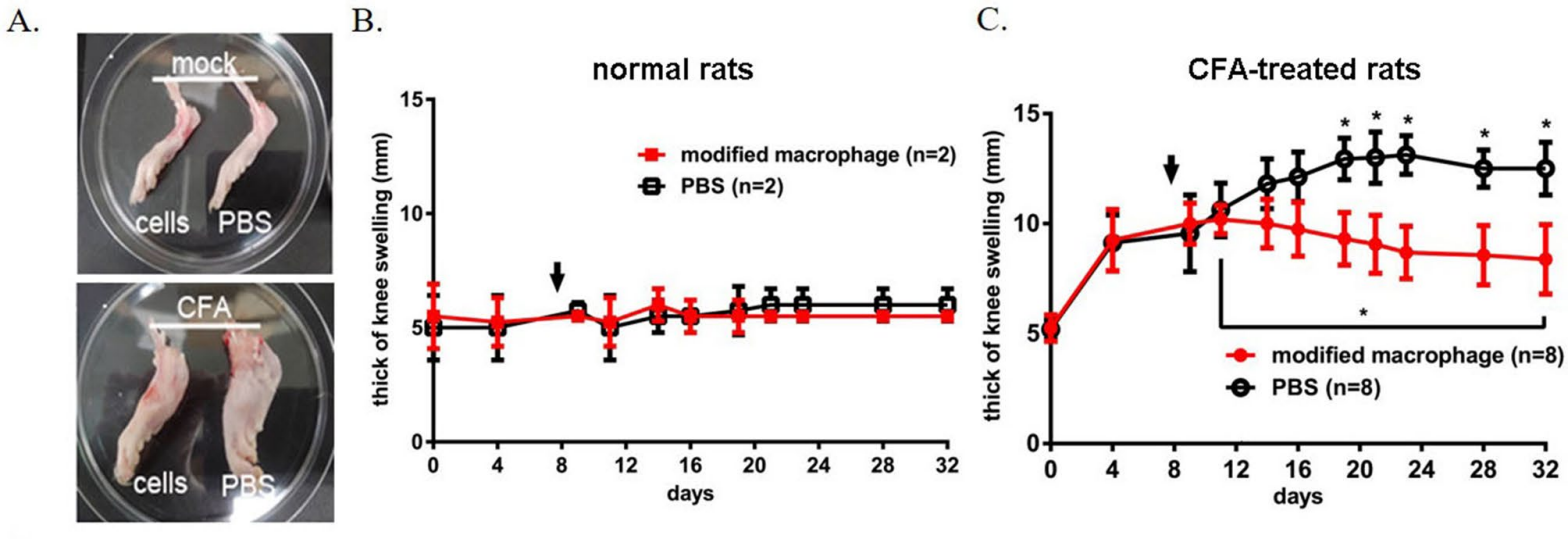
The therapeutic effect of the modified macrophages on arthritic joints. The up panel A shows the hind knee joints of a rat without arthritis, treated with modified macrophages (cells) or without cells (PBS). The down panel A shows the hind knee joints of rats with adjuvant (CFA)-induced arthritis, treated with cells or without cells (PBS). The thickness of knee swelling in rats without arthritis (**B**) or with arthritis (**C**) was measured after the treatments with the modified macrophages or (PBS). Arrow, the time of cell transplantation. *, *p* < 0.05, one-way ANOVA.

**Figure 7:**
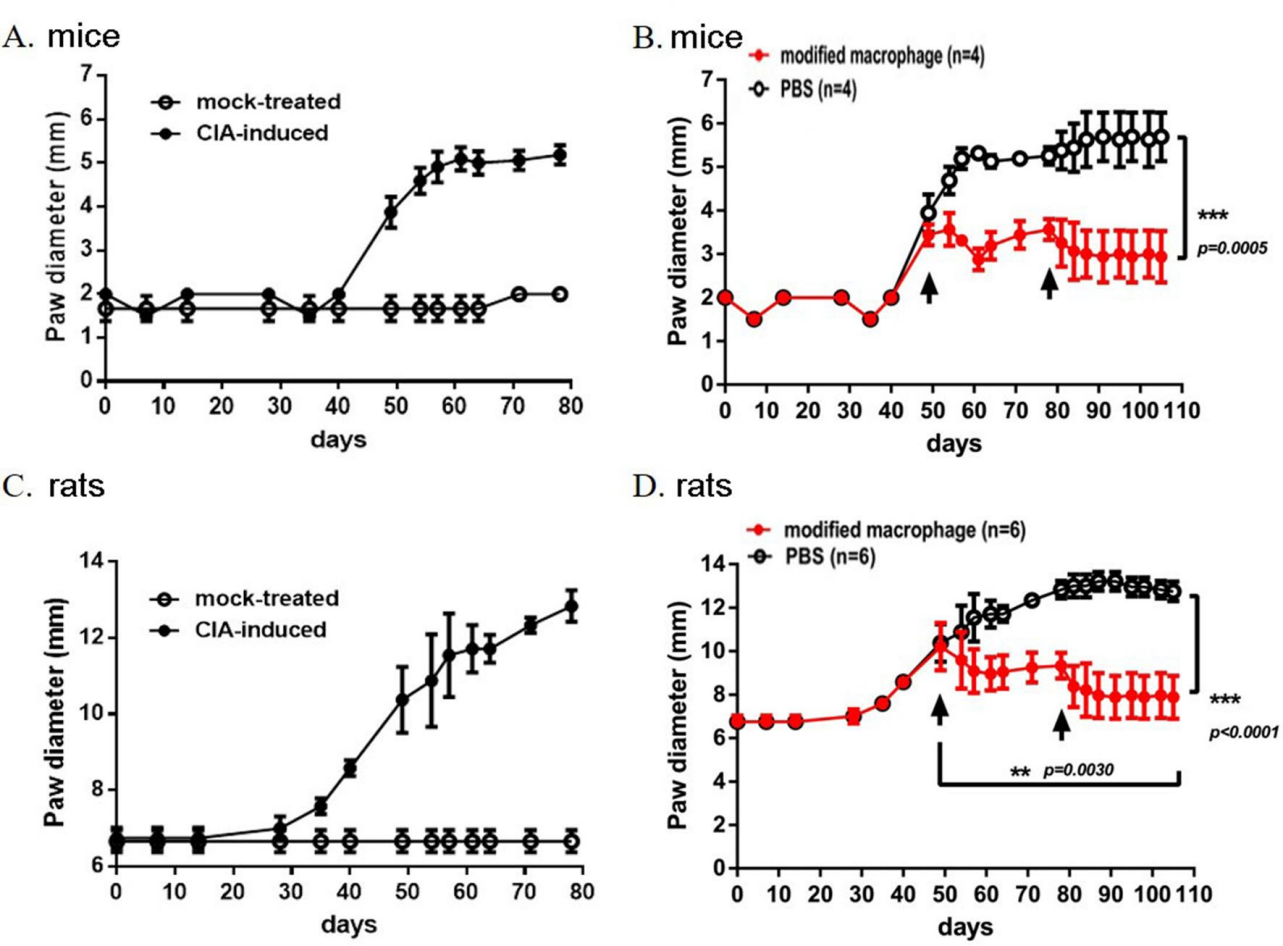
The C57BL/6 mice (**A**, **B**) and SD rats (**C**, **D**) with collagen-induced arthritis were treated with (**B**, **D**) or not treated with (**A**, **C**) the modified macrophages. The thickness of knee swelling in the rodents was measured, and the days of cell transplantation were indicated as arrows. **, *p* < 0.01; ***, *p* < 0.001, one-way ANOVA.

CD206^+^ M2 macrophages are featured with anti-inflammatory activities, and CD206^+^/CD14^+^/Col II^+^ modified macrophages may exhibit similar potency for preventing inflammation. We investigated the therapeutic potential of the modified macrophages on arthritis. We established inflammatory arthritis in rats by administration of complete Freund’s adjuvant (CFA) in hindlimb joints. The RA model in both mice and rats was also established by injection of collagen into the tail vein. Significant joint and paw swelling were observed on day 7 and day 40 after the CFA and collagen treatment, respectively. The gross pathology persisted for 3 months if no intervention was introduced. On day 7 and day 50 after CFA and collagen induction, respectively, the rodents were intra-articularly injected with 0.2 mL PBS (mock) or with 2 × 10^5^ M-CSF/IL-4-treated PBMNCs (Figs. 6 and 7).

We measured the diameter of each knee joint or paw every 2–3 days post-treatment. Treating the normal rats with PBS and modified macrophages showed no attenuation effect on joint swelling (Fig. 6B). Importantly, providing M-CSF/IL-4-treated PBMNCs significantly reduced the progression and the severity of the CFA-induced arthritis, compared to the joints on day 7 (*p* = 0.016, *p* < 0.05) and the PBS-treated joints on day 32 (*p* = 0.017, *p* < 0.05) (Fig. 6C). Similar results were observed in the collagen-induced arthritis model in mice (Fig. 7A,B) and rats (Fig. 7C,D). The inhibition of the arthritis could not be mediated by the soluble polarization factors due to the complete removal of the original medium by twice washes. Notably, the slight recurrence of tissue inflammation in modified macrophage-treated RA rodents was noticed on day 70 after the first cell administration on day 50 (Fig. 7B and D). A secondary cell transplantation boost on day 80 significantly attenuated the paw swelling till day 105, and no sign of recurrence was observed. These results suggest that the modified macrophages might be therapeutically effective for controlling OA or RA inflammation.

## Discussion

Monocytes have multiple functions and can differentiate into several distinct lineages, such as macrophage and dendritic cells. Derivative M1 and M2 macrophages carry out pro- or anti-inflammatory responses, respectively. Dendritic cells in tissues are the primary antigen-presenting cells (APC) to trigger T cell recognition and activation. In addition to the roles of immune modulation, macrophages and local fibroblasts can promote tissue repair at inflammatory sites. In this study, we discovered that monocytes could become a novel population, featured with Col II expression, induced by high-dose M-CSF or GM-CSF. This unique cell might exhibit both anti-inflammatory and tissue-repairing properties to ameliorate the destructive progress of arthritis in patients.

In general, monocytes/macrophages are supposed to secret cytokines and promote collagen formation in the inflammatory tissues, including both Col I and Col III, for tissue repairing and immunomodulation^15,16^. Notably, a recent study revealed that not only myofibroblast cells in the damaged tissues, but also blood monocyte-derived macrophages directly contribute to Col I deposit and scar formation during mouse heart repair^17^. This novel result highlights that monocytes/macrophages orchestrate the cellular properties of migration, invasion, and tissue reconstruction. In this study, treating the monocytes with both M-CSF and GM-CSF steered about 40–50% adherent PBMNCs (Fig. 1), and 10–20% suspended PBMNCs (Fig. 2) to express Col II. Higher Col II expression seems correlative to the M-CSF and adhesion-triggered maturation in monocyte-derived cells. Whether the down-regulation of Col II prevents the monocyte maturation or migration remains unclear. The maturation of the Col II and the extracellular seretion of the Col II need further investigation. It is also interesting to examine the role of Col II and its reciprocal relationship with Col I, Col III and other ECM in the cell migration or tissue repairment of modified macrophages.

Col II is specifically expressed by chondrocytes and is the major matrix protein in cartilage. In addition to the cartilage, Col II is also detected in the vitreous body of eyes, accounting for 60–75% of the total collagen proteins^18^. Non-cartilage expression of Col II, especially for the mRNA of IIA splicing form, is also found in developmental paraxial mesodermal cells, such as somites and skeletal primordial embryonic structure^18^. The spatial and temporal regulation of Col II in non-cartilage tissue is largely obscure. It is interesting to explore whether the non-cartilage Col II expression in modified macrophages and in embryonic paraxial mesoderm share similar core regulatory complexes and what novel regulators are required for the ex vivo enforced Col II expression.

Sox9, a member of the family of SRY (Sex determining region of the Y chromosome)-related high-mobility-group box (SOX) transcription factors, is a dominant factor for the Col II expression and cartilage formation^19^. Human *SOX9* gene mutations contribute to campomelic dysplasia, a severe skeletal malformation syndrome with gonadal dysgenesis, indicating that SOX9 is a pivotal factor in controlling bone and testes development^19^. Whether Sox9 participates in the Col II expression in the modified macrophages remains unclear. Regarding the signaling pathway of M-CSF and Sox9 activation, M-CSF activation triggers classical receptor tyrosine kinases to activate intracellular MAPK, PI3K, and Stat3 pathway and to promote macrophage proliferation, differentiation, and survival^20^. Stat3 signal is essential to activate Sox9 expression by binding to the proximal promoter region of *Sox9* genome^21^. It is possible that after the treatment of extra-physiological M-CSF/GM-CSF stimuli on the cell membrane, intracellular phosphorylated Stat3 triggers the Sox9 induction and Sox9-downstream Col II gene activation. Interestingly, a recent finding indicated that RAW264.9 cells and mouse bone marrow-derived macrophages are competent to activate *Sox9* genes after IL-4 treatment^22^. However, their Col II protein induction in the macrophages was not investigated.

Notably, Sox9 is a well-characterized chromatin modulator and usually binds to the super-enhancer to control the lineage status, cellular stemness, and differentiation^19^. Sox9 and its associated transcriptional regulators, such as Sox5 and Sox6, are master regulators for the spatiotemporal expression of Col II^19^ and might coordinately participate in the Col II expression in the modified macrophages. The genome-level regulatory elements of *Col IIα1*, such as the intron 1 enhancer and intron 6 enhancers, and their epigenetic modification on the ectopic Col II expression may also involve the Sox9-mediated Col II expression of the modified macrophages^23,24^.

In OA and RA patients, the destructive cartilage and joint lining cells usually bring elevated inflammatory cytokines, polarized M1 macrophages, and synovitis during the disease progress. The current candidates of cell products for the disease-modifying OA drugs (DMOADs) include the mesenchymal stem cells, TGF-β expressing 293 cells and bone marrow-derived mononuclear cells to ameliorate the inflammation and promote tissue regeneration^25,26^. Here, we provide a new candidate for DMOADs, modified macrophages, featured with both CD206 and Col II matrix expression to exhibit both anti-inflammatory and tissue repair activity. The Col II is crucial for the reconstruction of hyaline cartilage. A clinical trial demonstrated that intra-articular injection of bovine Col II showed pain-relief efficacy, promising immune suppression, and increased glycosaminoglycan production from the endogenous chondrocytes^27^. Interestingly, a recent study also revealed that squid Col II could relieve OA symptoms by inhibiting the cell death of chondrocytes and promoting the M2 macrophage polarization^28^. These findings emphasize the importance of Col II supplement in controlling arthritis progress. The discovery of the Col II-expressing modified macrophages, and the amelioration of arthritis diseases in rodents provide promising preclinical evidence bases for future clinical study of the cells in controlling arthritis.

## Materials and methods

### The blood samples and polarization of the mononuclear cells

Peripheral blood samples were collected from donors and stored in a collecting tube with EDTA or heparin, approved by the Research Ethics Committee of Chinese Medical University and Hospital in Taichung, Taiwan (CMUH109-Rec1-012) and carried out in Asia University Hospital, Taichung, Taiwan. All methods were performed following the guidelines and regulations of the approved protocols by the Research Ethics Committee. Informed consents were obtained from all subjects who agreed to join this project. Twenty mL of the peripheral blood sample was carefully loaded on the 20 mL Ficoll-Paque premium interface. After centrifuging at 700 × g for 15 min, the cells in the upper layer of the tube (i.e., peripheral blood mononuclear cells, PBMNCs) were transferred to 50 mL tubes. The cell pellet was obtained after centrifuging at 500 × g for 5 min, and the percentage of monocyte in the cell pellet was measured by flow cytometry (Accuri, Becton–Dickinson, USA) using anti-CD14 FITC conjugated antibody (BioLegend, USA). In general, 1–3 × 10^7^ PBMNCs can be harvested from 20 mL blood.

The isolated PBMNCs (including lymphocytes, monocytes, and NK cells) were further incubated with M-CSF (R&D Systems, USA), GM-CSF (Peprotech, USA), or G-CSF (Peprotech, USA) in a basal medium containing RPMI-1640 medium (Thermo-Fisher, USA) with 2% human platelet lysates (Compass, USA) for indicated days on low-binding culture dishes (Alpha Plus Scientific, Taiwan) or 2.5 μg/cm^2^ fibronectin (STEMCELL Technologies)-coated slides. The other cytokines used for the Col II induction, such as IL-4, IL-10, IL-13 and TGF-β, were all obtained from R&D Systems.

### The flow cytometry analysis and immunocytostaining

The PBMNCs, cultured with 2% human platelet lysates and cytokines in a low-binding dish, were harvested at indicated times for immunocytostaining. The living membrane-intact cells were treated with CD14, CD206, and anti-Col II antibodies to determine the cell fate and protein expression on the cell membrane.

Adherent monocyte-derived macrophages were fixed on fibronectin-coated glass slides with a paraformaldehyde buffer. After the treatment with an intracellular staining permeabilization wash buffer (BioLegend), the cells were stained with the anti-human Col II antibodies (Abs), including rabbit polyclonal (AB761, Millipore) and mouse monoclonal Abs (MA5-12,789, Invitrogen, USA) for 1 h. Col II expressing cells were visualized with green Alexa Fluor 488-conjugated goat anti-rabbit IgG antibody (A11008, Invitrogen) or anti-mouse IgG antibody (31,569, Invitrogen). The total cell number was estimated by the 4',6-diamidino-2-phenylindole (DAPI) staining in the nuclei (blue).

### The RT and real-time PCR

The 1 × 10^8^ PBMNCs were cultured with a combination of 100 ng/mL M-CSF100 ng/mL IL-4 or PBS at 0.5, 1, 2, 4, 6 h in low-binding dishes (Alpha Sciences, Taiwan). The monocytes amongst the cultured PBMNCs were sorted with an Easysep selection kit containing anti-CD14 antibody on magnetic beads (Stemcell Technology, Vancouver, Canada), according to the manufacturer’s instrument. The purity of the monocytes was analyzed by flow cytometry with FITC conjugated anti-CD14 monoclonal antibody (BioLegend).

The total RNA of CD14^+^ cells was extracted by RNeasy mini kit (Qiagen) according to manufacturer’s recommendation. Reverse transcribed into first-strand cDNA using the Moloney murine leukemia virus reverse transcription kit (M-MuLV RT kit, Protech Technology Enterprise, Taiwan). Total RNA was initially denatured at 65 °C for 5 min and quickly placed on ice for at least 1 min. The annealing with oligo (dT)_18_ and random hexamers were conducted at 25 °C for 10 min, and the cDNA was synthesized at 42 °C for 60 min. The quantitative Col II mRNA expression levels were analyzed by the real-time reverse transcriptase-polymerase chain reaction (RT-PCR) (QuantStudio™ Pro 6 System, Thermo Fisher Scientific). The RNA expression level of Col II was normalized to that of glyceraldehyde 3-phosphate (GAPDH), and the fold differences were compared to the PBS control. The forward primer and reverse primer of Col II were 5’- CCC TGA GTG GAA GAG TGG AG -3’, and 5’- GAG GCG TGA GGT CTT CTG TG -3’, respectively. The primers for GAPDH were forward 5’- TCG ACA GTC AGC CGC ATC TTC TTT -3’, reverse 5’- ACC AAA TCC GTT GAC TCC GAC CTT -3’. The real-time PCR applied SYBR green dye with low rox concentration (PowerUP SYBR green master mix, Thermo Fisher Scientific). Each sample was initially denatured at 95 °C for 10 min and then underwent 40-cycles reactions, including denaturation at 95 °C for 15 s, annealing and extension at 60 °C for 1 min.

### Animal experiments

The in vivo evaluation of the effect of modified macrophages in rodents was approved by the Institutional Animal Care and Use Committee (IACUC) of National Chung Hsing University (NCHU) (NCHU 109–093) in Taichung, Taiwan and carried out in the Department of Life Sciences NCHU, Taichung, Taiwan. Experiments were performed in accordance with Taiwan Animal Protection Act and in compliance with the ARRIVE guidelines. Animals were housed and maintained at room temperature under a typical light cycle environment. The food and water were provided ad libitum.

Adult male Sprague–Dawley (SD) rats, weighing 200–250 g, were housed in a room with constant temperature (24–26 ℃) and humidity (40–60%) and had free access to food and water. A 0.25 mL of complete Freund’s adjuvant (CFA, Sigma-Aldrich, USA) was injected into the hind knee joint of each rat to induce inflammatory arthritis. On day 6, the inflammation was boosted by injecting an additional 0.05 mL of CFA into the same sites. On Day 7, when the arthritis was established, the arthritic knee joints were treated with 0.2 mL of PBS (control) or 2 × 10^5^ M-CSF/IL-4-induced modified macrophages in 0.2 mL PBS. Non-binding cytokines to the cells were cleared by PBS wash twice. Knee joints were measured using a vernier every 2–3 days for two weeks.

Collagen-induced arthritis (CIA) in rodents is a classical model of human rheumatoid arthritis (RA). CIA was successfully established in both C57BL/6 mice and SD rats via the subcutaneous injection of type II collagen (200 μg) into the tail vein. Significant paw swelling was observed on day 40 and persisted to day 105. The 2 × 10^5^ modified macrophages were intra-articularly (IA) injected into the left knee of each mouse or rat on day 50 (1^st^ dose) and day 80 (2^nd^ dose). The PBS (phosphate buffer saline) was IA-injected into the right knee of each mouse or rat as a mock-treatment control. Knee joints were measured using a vernier every 2–3 days for two weeks.

### Statistic analysis

We used a Student *t*-test or one-way ANOVA to determine the significance of differences between the experimental groups. This study's graphics creation and statistical analysis were conducted using Microsoft Excel (version 2019) or GraphPad Prism 9 (GraphPad, La Jolla, CA, USA).

## Data Availability

The data that support the findings of this study are available from Hong-Lin Su upon request.
